# Supplementary figures and images for: From trace to trace maker: Oligocene–Miocene coprolites of southern Poland and their potential producers
Source: PeerJ. 2025 Nov 3;13:e20242. doi: 10.7717/peerj.20242 (PMC12591054; doi:10.7717/peerj.20242)

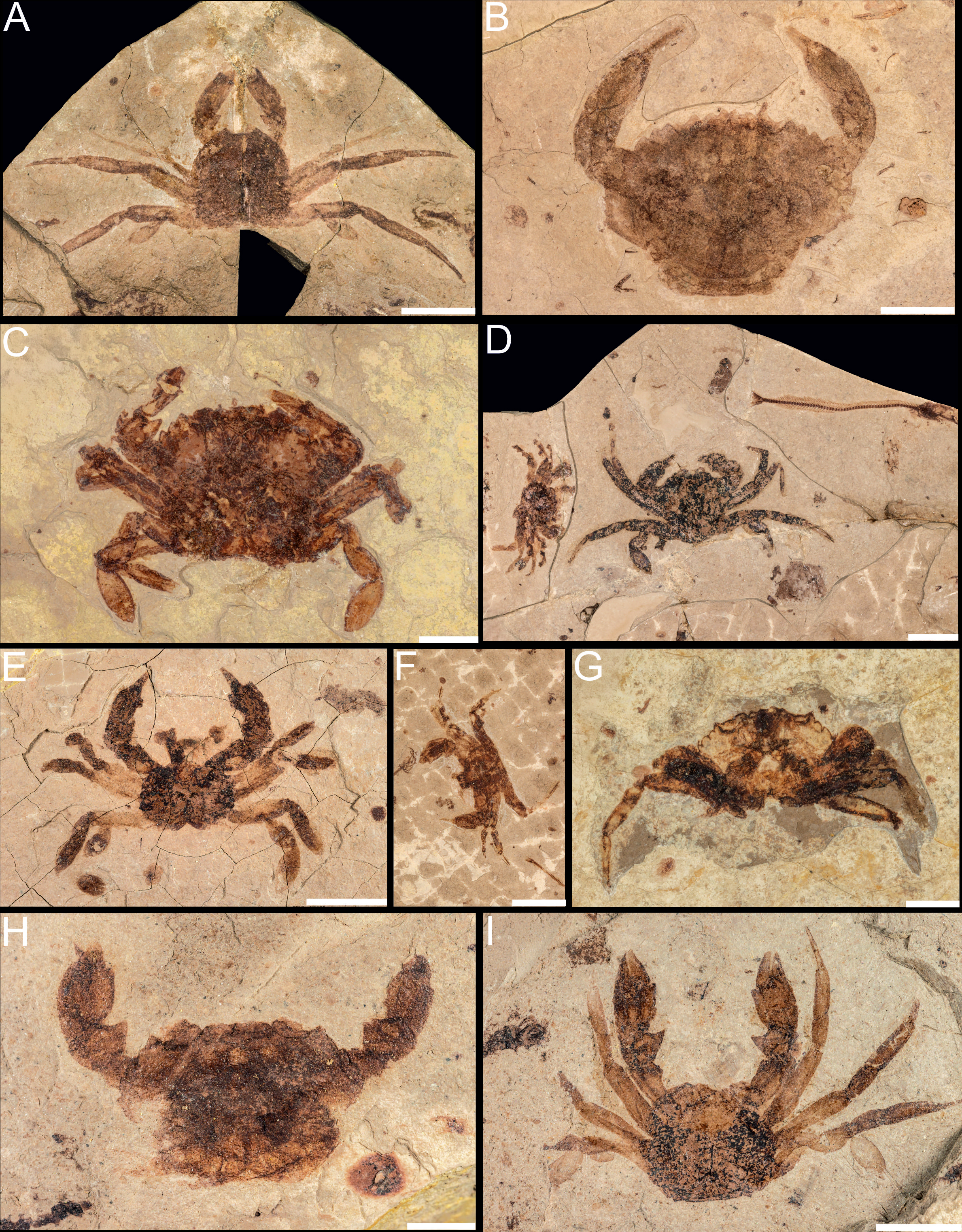

Supplement: Supplemental Information 1 — (A) Kr.J-7. (B) Kr.H-1. (C) Kr.JR-2. (D) Kr.J-3. (E) Kr.J-11. (F) Kr.J-16. (G) Kr.J-12. (H) Kr.J-6. (I) Kr.J-3. Scale bar equals one cm. [file peerj-13-20242-s001.jpg]

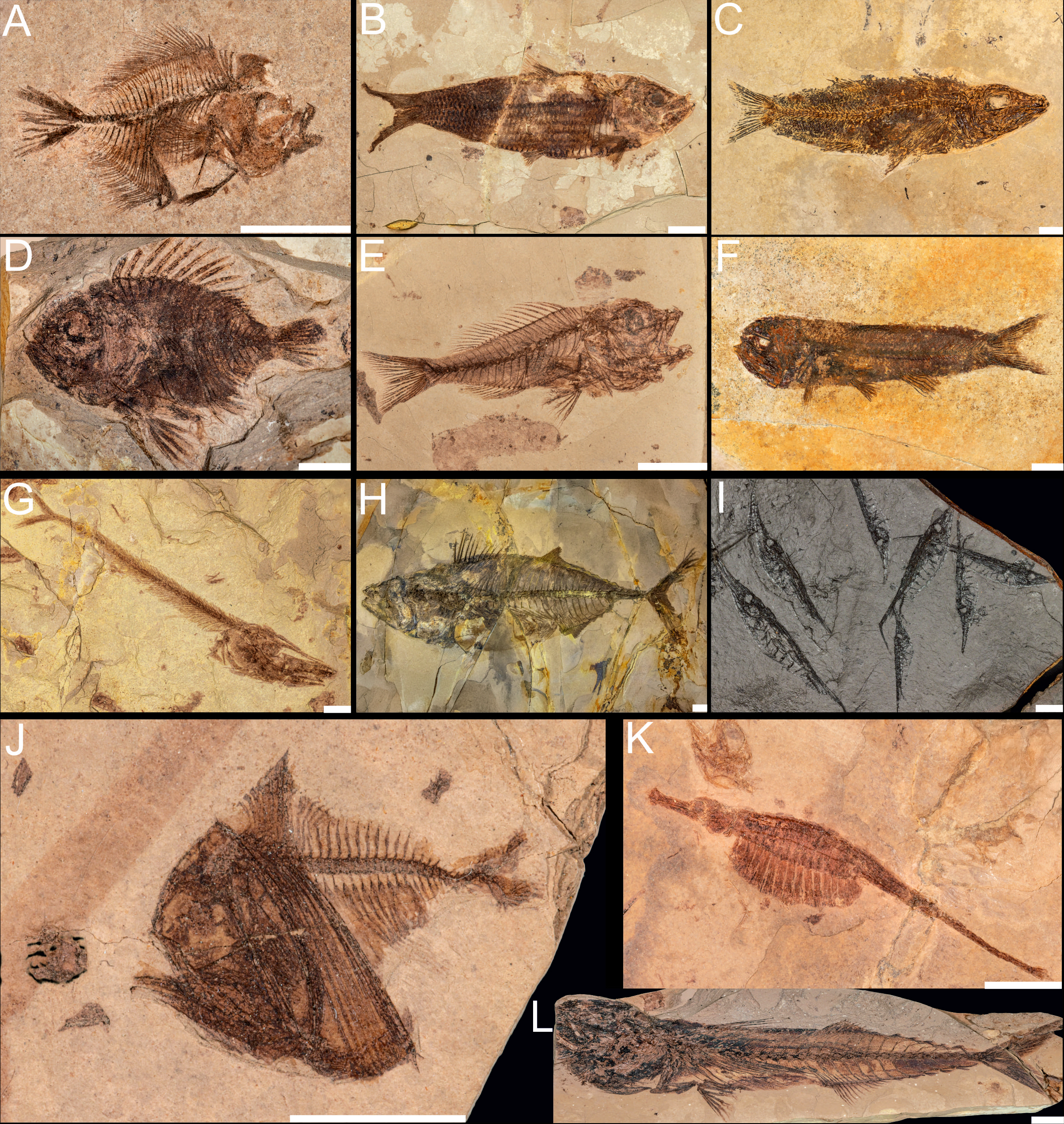

Supplement: Supplemental Information 2 — (A) Specimen representing u nidentified taxa, Ma 31. (B) Clupea sp., ROJ-215. (C) Specimen representing u nidentified taxa, ROJ-212. (D) Specimen representing u nidentified taxa, ROL-305. (E) Specimen representing u nidentified taxa, ROJ-307. (F) Eomyctophum sp., Ma-52. (G) Holosteus sp., ROJR-170. (H) Unidentified taxa of Scombridae family, ROL-47. (I) Centriscus sp., ROJ-514. (J) Argyropelecus sp., ROL-221. (K) Hipposyngnathus sp., ROJ-211. (L) Specimen representing u nidentified taxa, ROL-328. Scale bar equals 1 cm. [file peerj-13-20242-s002.jpg]

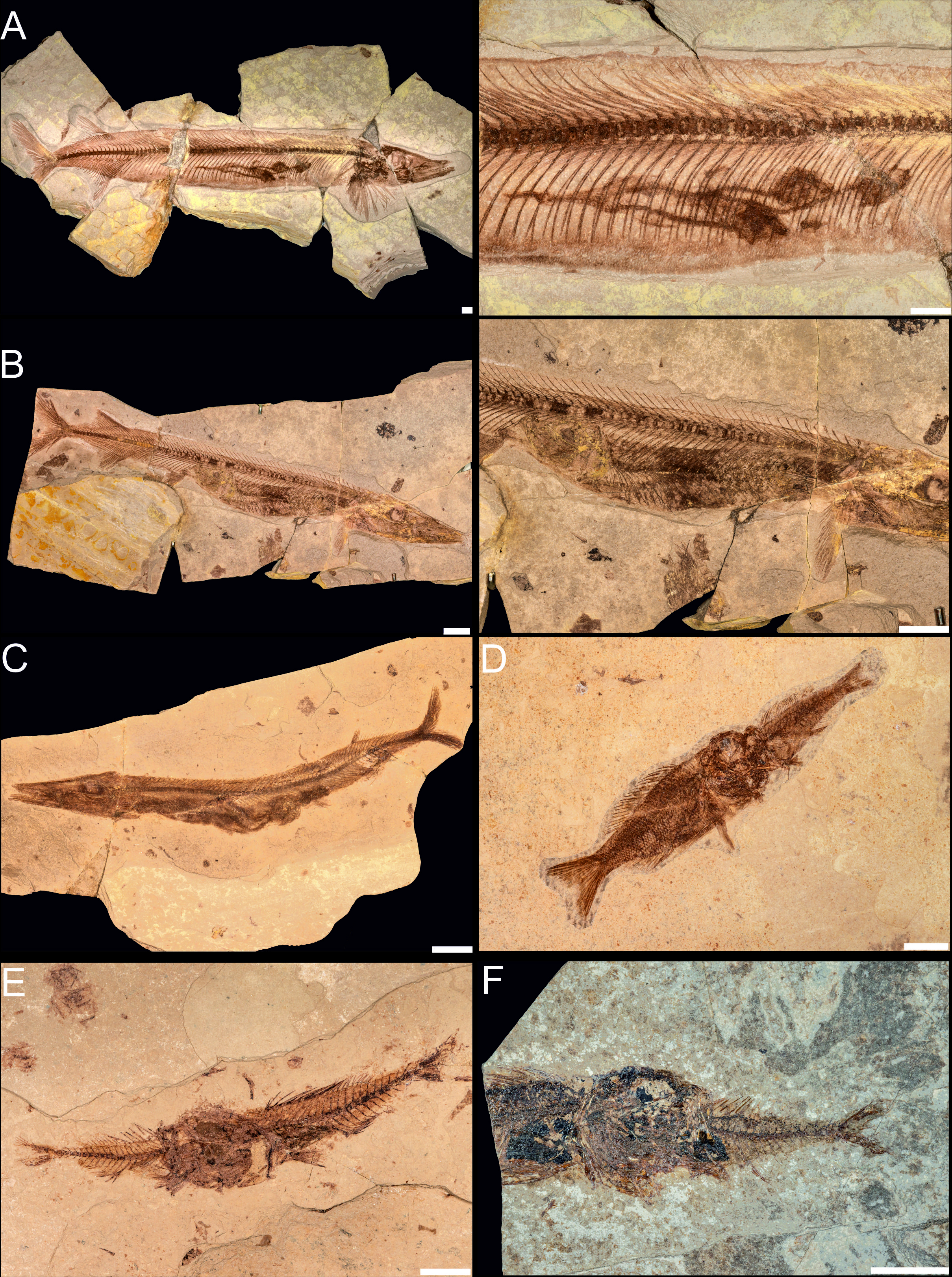

Supplement: Supplemental Information 3 — (A) Holosteus sp., ROJ-17. (B) Holosteus sp., ROJ-22. (C) Holosteus sp., ROJ-45. (D) Oligoserranoides sp., ROR-153. (E) Oligoserranoides sp., ROJ-47. (F) Oligoserranoides sp., RORR-7. Scale bar equals 1 cm. [file peerj-13-20242-s003.jpg]

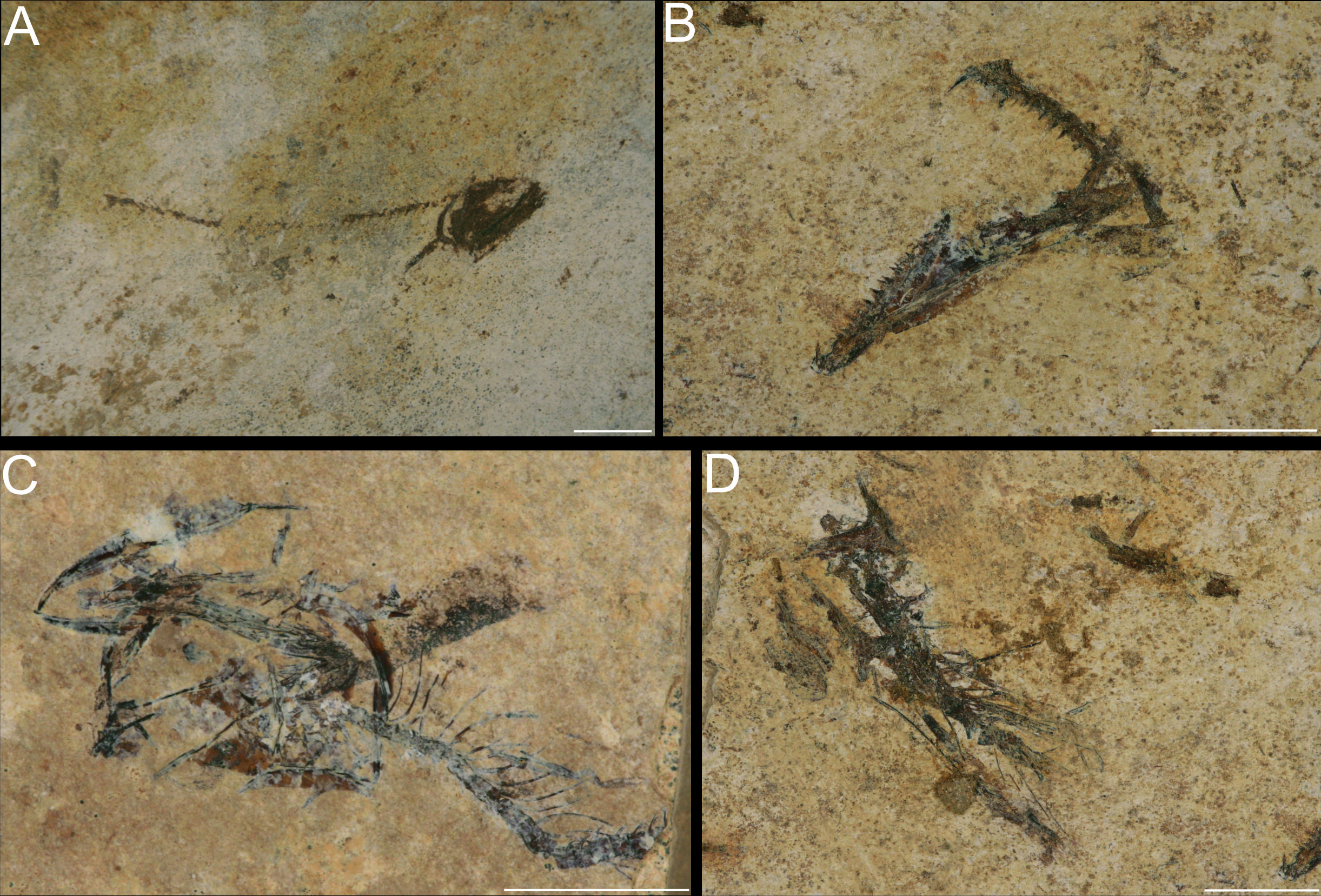

Supplement: Supplemental Information 4 — (A) Scopeloides sp. GIUS10–3796/O/F1. (B) Jaw of Lepidopus sp. (C, D) probably Scopeloides sp. GIUS10–3796/O/F3, 4. Scale bar equals 1 cm. [file peerj-13-20242-s004.jpg]

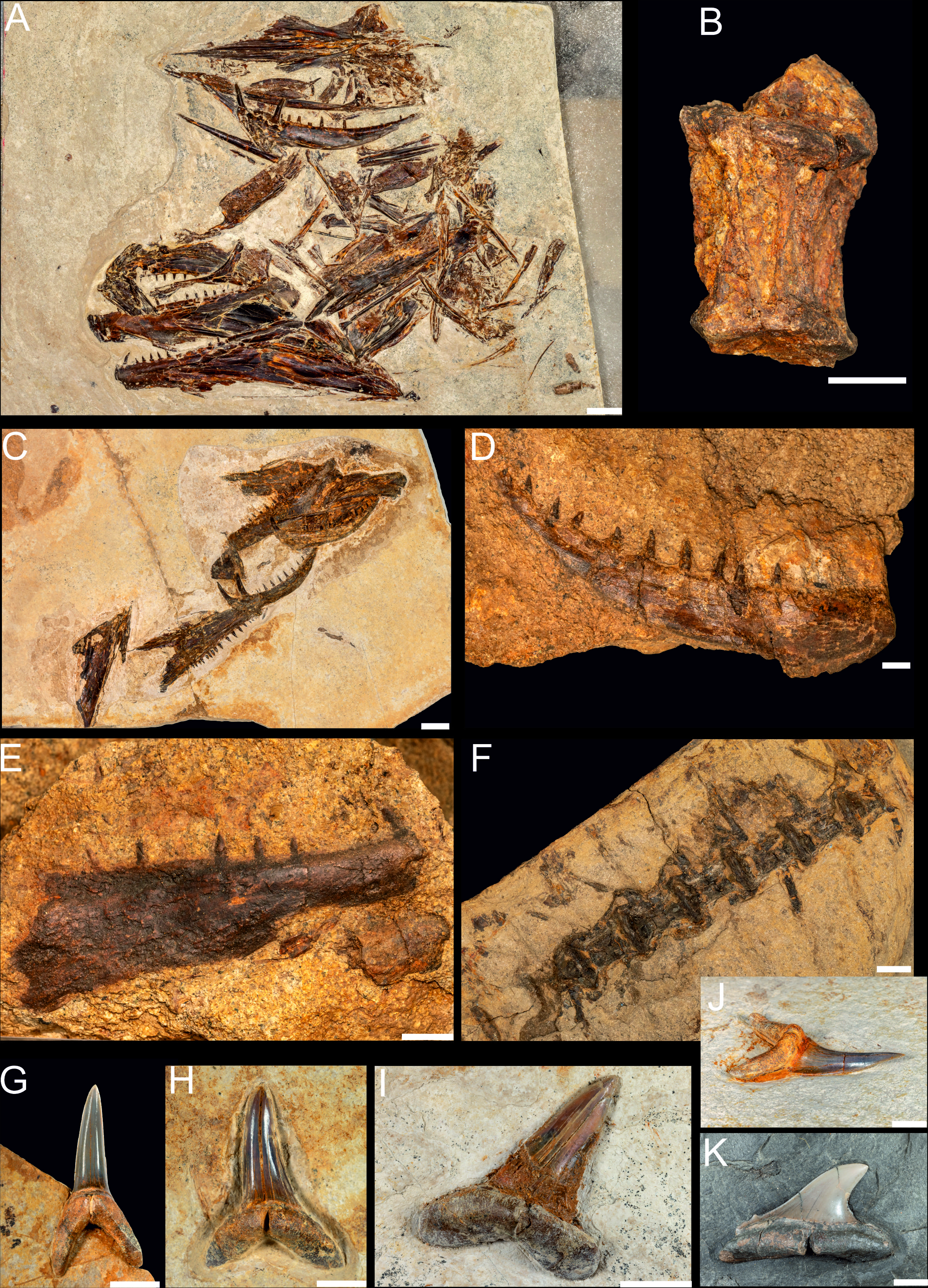

Supplement: Supplemental Information 5 — (A) Lepidopus sp., ROU-400. (B) Lepidopus sp., ROU-405. (C) Lepidopus sp., Ma-5. (D) Lepidopus sp., ROU-40. (E) Lepidopus sp., ROU-42. (F) Lepidopus sp., ROL-55. (G) Isurus sp., ROM-ZR-100.(H) Isurus sp., ROM-ZR-103. (I) Isurus sp., ROM-ZR-107. (J) Isurus sp., ROM-ZR-112. (K) Isurus sp., ROJ-ZR-123. Scale bar equals 1 cm. [file peerj-13-20242-s005.jpg]

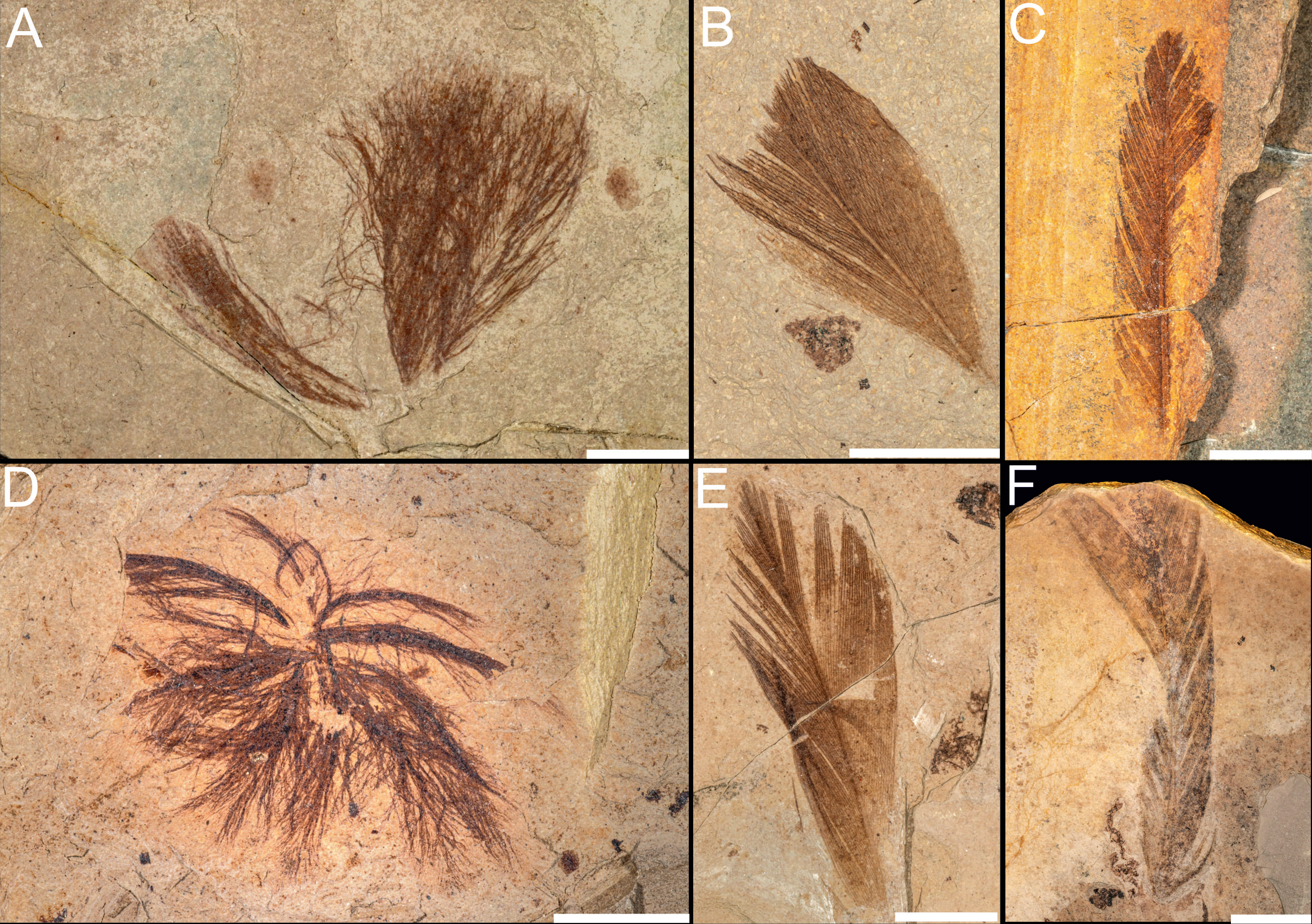

Supplement: Supplemental Information 6 — (A) MSMD.Av. Jam-11. (B) MSMD.Av. Jam-14. (C) MSMD.Av. S.Bir-3. (D) MSMD.Av. Jam-1. (E) MSMD.Av. J.Ros-9. (F) MSMD.Av. Jam-15. Scale bar equals 1 cm. [file peerj-13-20242-s006.jpg]

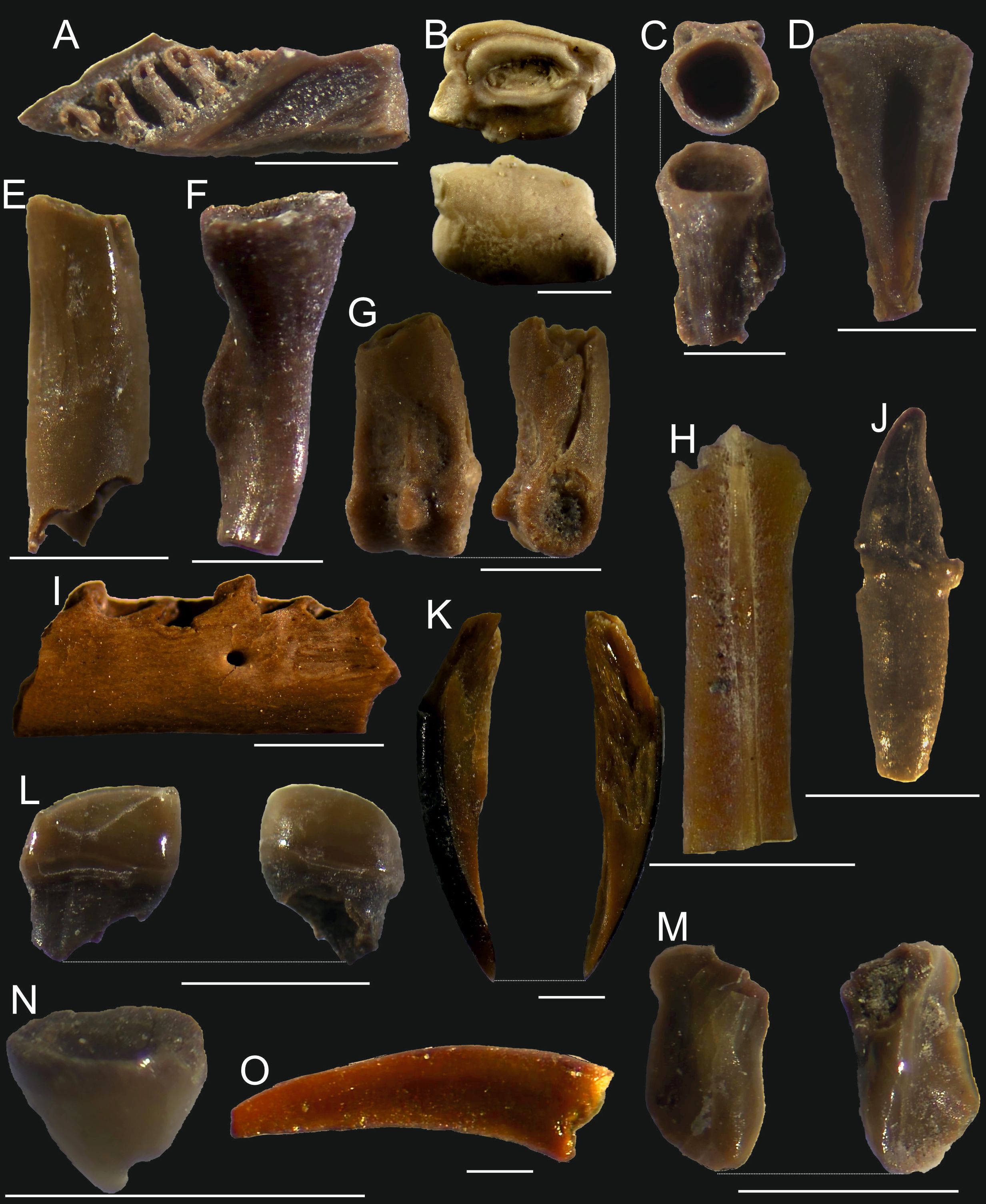

Supplement: Supplemental Information 7 — (A) Jaw of a Lacertidae lizard. (B) Otolith of Klingobius andjelkocae. (C, D) Vertebrae of indeterminated rodents. (E–H) Bones of indeterminate vertebrates. (I) Jaw of a rodent. (J) Tooth of Chiroptera. (K) Incisor of Castocrinae. (L–N) Talpidaeteeth. (O) Tooth of an unidentified predator. Scale bar equals 1 mm. [file peerj-13-20242-s007.jpg]
