# Supplementary material for: From trace to trace maker: Oligocene–Miocene coprolites of southern Poland and their potential producers
Source: PeerJ. 2025 Nov 3;13:e20242. doi: 10.7717/peerj.20242 (PMC12591054; doi:10.7717/peerj.20242)
Supplement: Supplemental Information 8 [file peerj-13-20242-s008.docx]

**Table 1:**

**Oligocene coprolite list.**

| **Specimen** | **Dimensions (mm)** | **Shape** | **Age** | **Site** |
| --- | --- | --- | --- | --- |
| GIUS 10–3796/O/1 | 31x5 | Sinusoidal | Oligocene - Rupelian | Menilite-Krosno Series (M-KS)- Kąkolówka I |
| GIUS 10–3796/O/2 | 20x3 | Curved, Fig. 2a | Oligocene - Rupelian | M-KS-Kąkolówka I |
| GIUS 10–3796/O/3 | 22x5 | Elongated | Oligocene - Rupelian | M-KS-Kąkolówka I |
| GIUS 10–3796/O/4 | 14x14 | Oval | Oligocene - Rupelian | M-KS-Kąkolówka I |
| GIUS 10–3796/O/5 | 17x4 | Sinusoidal | Oligocene - Rupelian | M-KS-Kąkolówka I |
| GIUS 10–3796/O/6 | 18x7 | Curved | Oligocene - Rupelian | M-KS-Kąkolówka I |
| GIUS 10–3796/O/7 | 18x3 | Curved, Fig.2b | Oligocene - Rupelian | M-KS-Kąkolówka I |
| GIUS 10–3796/O/8 | 25x10 | Elongated | Oligocene - Rupelian | M-KS-Kąkolówka I |
| GIUS 10–3796/O/9 | 23x8 | Elongated | Oligocene - Rupelian | M-KS-Kąkolówka I |
| GIUS 10–3796/O/10 | 38x9 | Sinusoidal | Oligocene - Rupelian | M-KS-Kąkolówka I |
| GIUS 10–3796/O/11 | 24x19 | Oval | Oligocene - Rupelian | M-KS-Kąkolówka I |
| GIUS 10–3796/O/12 | 17x9 | Curved | Oligocene - Rupelian | M-KS-Kąkolówka I |
| GIUS 10–3796/O/13 | 23x6 | S-shaped | Oligocene - Rupelian | M-KS-Kąkolówka I |
| GIUS 10–3796/O/14 | 29x8 | Sinusoidal | Oligocene - Rupelian | M-KS-Kąkolówka I |
| GIUS 10–3796/O/15 | 35x11 | Curved | Oligocene - Rupelian | M-KS-Kąkolówka I |
| GIUS 10–3796/O/16 | 38x10 | Sinusoidal | Oligocene - Rupelian | M-KS-Kąkolówka I |
| GIUS 10–3796/O/17 | 41x9 | Elongated | Oligocene - Rupelian | M-KS-Kąkolówka I |
| GIUS 10–3796/O/18 | 12x2 | Sinusoidal | Oligocene - Rupelian | M-KS-Kąkolówka I |
| GIUS 10–3796/O/19 | 25x6 | Curved | Oligocene - Rupelian | M-KS-Kąkolówka I |
| GIUS 10–3796/O/20 | 28x14 | Irregular | Oligocene - Rupelian | M-KS-Kąkolówka I |
| GIUS 10–3796/O/21 | 15x12 | Oval | Oligocene - Rupelian | M-KS-Kąkolówka I |
| GIUS 10–3796/O/22 | 17x5 | Elongated | Oligocene - Rupelian | M-KS-Kąkolówka I |
| GIUS 10–3796/O/23 | 25x2 | Sinusoidal, Fig.2c | Oligocene - Rupelian | M-KS-Kąkolówka I |
| GIUS 10–3796/O/24 | 27x5 | S-shaped | Oligocene - Rupelian | M-KS-Kąkolówka I |
| GIUS 10–3796/O/25 | 25x7 | Sinusoidal | Oligocene - Rupelian | M-KS-Kąkolówka I |
| GIUS 10–3796/O/26 | 14x4 | Curved | Oligocene - Rupelian | M-KS-Kąkolówka I |
| GIUS 10–3796/O/27 | 17x5 | Elongated | Oligocene - Rupelian | M-KS-Kąkolówka I |
| GIUS 10–3796/O/28 | 18x17 | Oval | Oligocene - Rupelian | M-KS-Kąkolówka I |
| GIUS 10–3796/O/29 | 19x6 | Sinusoidal | Oligocene - Rupelian | M-KS-Kąkolówka I |
| GIUS 10–3796/O/30 | 25x15 | Irregular | Oligocene - Rupelian | M-KS-Kąkolówka I |
| GIUS 10–3796/O/31 | 24x11 | Sinusoidal | Oligocene - Rupelian | M-KS-Kąkolówka I |
| GIUS 10–3796/O/32 | 39x9 | Elongated | Oligocene - Rupelian | M-KS-Kąkolówka I |
| GIUS 10–3796/O/33 | 26x19 | Sinusoidal | Oligocene - Rupelian | M-KS-Kąkolówka I |
| GIUS 10–3796/O/34 | 15x6 | Curved | Oligocene - Rupelian | M-KS-Kąkolówka I |
| GIUS 10–3796/O/35 | 22x7 | S-shaped | Oligocene - Rupelian | M-KS-Kąkolówka I |
| GIUS 10–3796/O/36 | 30x28 | Irregular | Oligocene - Rupelian | M-KS-Kąkolówka I |
| GIUS 10–3796/O/37 | 14x10 | Irregular | Oligocene - Rupelian | M-KS-Kąkolówka I |
| GIUS 10–3796/O/38 | 28x11 | Sinusoidal | Oligocene - Rupelian | M-KS-Kąkolówka I |
| GIUS 10–3796/O/39 | 34x10 | Curved | Oligocene - Rupelian | M-KS-Kąkolówka I |
| GIUS 10–3796/O/40 | 22x9 | Elongated | Oligocene - Rupelian | M-KS-Kąkolówka I |
| GIUS 10–3796/O/41 | 27x6 | Sinusoidal | Oligocene - Rupelian | M-KS-Kąkolówka I |
| GIUS 10–3796/O/42 | 32x14 | S-shaped | Oligocene - Rupelian | M-KS-Kąkolówka I |
| GIUS 10–3796/O/43 | 25x12 | Curved | Oligocene - Rupelian | M-KS-Kąkolówka I |
| GIUS 10–3796/O/44 | 16x5 | Sinusoidal | Oligocene - Rupelian | M-KS-Kąkolówka I |
| GIUS 10–3796/O/45 | 34x21 | Irregular | Oligocene - Rupelian | M-KS-Kąkolówka I |
| GIUS 10–3796/O/46 | 37x12 | Elongated | Oligocene - Rupelian | M-KS-Kąkolówka I |
| GIUS 10–3796/O/47 | 39x9 | Sinusoidal | Oligocene - Rupelian | M-KS-Kąkolówka I |
| GIUS 10–3796/O/48 | 14x13 | Oval | Oligocene - Rupelian | M-KS-Kąkolówka I |
| GIUS 10–3796/O/49 | 17x7 | Elongated | Oligocene - Rupelian | M-KS-Kąkolówka I |
| GIUS 10–3796/O/50 | 33x9 | S-shaped | Oligocene - Rupelian | M-KS-Kąkolówka I |
| GIUS 10–3796/O/51 | 32x12 | Sinusoidal | Oligocene - Rupelian | M-KS-Kąkolówka I |
| GIUS 10–3796/O/52 | 30x14 | Curved | Oligocene - Rupelian | M-KS-Kąkolówka I |
| GIUS 10–3796/O/53 | 26x8 | Elongated | Oligocene - Rupelian | M-KS-Kąkolówka I |
| GIUS 10–3796/O/54 | 27x7 | Sinusoidal | Oligocene - Rupelian | M-KS-Kąkolówka I |
| GIUS 10–3796/O/55 | 24x21 | Irregular | Oligocene - Rupelian | M-KS-Kąkolówka I |
| GIUS 10–3796/O/56 | 39x12 | Sinusoidal | Oligocene - Rupelian | M-KS-Kąkolówka I |
| GIUS 10–3796/O/57 | 26x8 | Curved | Oligocene - Rupelian | M-KS-Kąkolówka I |
| GIUS 10–3796/O/58 | 29x17 | Irregular | Oligocene - Rupelian | M-KS-Kąkolówka I |
| GIUS 10–3796/O/59 | 37x13 | S-shaped | Oligocene - Rupelian | M-KS-Kąkolówka I |
| GIUS 10–3796/O/60 | 25x4 | Curved, Fig. 2f | Oligocene - Rupelian | M-KS-Kąkolówka I |
| GIUS 10–3796/O/61 | 32x12 | Curved | Oligocene - Rupelian | M-KS-Kąkolówka I |
| GIUS 10–3796/O/62 | 17x16 | Oval | Oligocene - Rupelian | M-KS-Kąkolówka I |
| GIUS 10–3796/O/63 | 28x9 | Sinusoidal | Oligocene - Rupelian | M-KS-Kąkolówka I |
| GIUS 10–3796/O/64 | 39x15 | Elongated | Oligocene - Rupelian | M-KS-Kąkolówka I |
| GIUS 10–3796/O/65 | 26x14 | Sinusoidal | Oligocene - Rupelian | M-KS-Kąkolówka I |
| GIUS 10–3796/O/66 | 27x13 | Curved | Oligocene - Rupelian | M-KS-Kąkolówka I |
| GIUS 10–3796/O/67 | 24x10 | Elongated | Oligocene - Rupelian | M-KS-Kąkolówka I |
| GIUS 10–3796/O/68 | 23x8 | Curved | Oligocene - Rupelian | M-KS-Kąkolówka I |
| GIUS 10–3796/O/69 | 18x11 | Irregular | Oligocene - Rupelian | M-KS-Kąkolówka I |
| GIUS 10–3796/O/70 | 19x7 | Sinusoidal | Oligocene - Rupelian | M-KS-Kąkolówka I |
| GIUS 10–3796/O/71 | 28x9 | S-shaped | Oligocene - Rupelian | M-KS-Kąkolówka I |
| GIUS 10–3796/O/72 | 35x14 | Sinusoidal | Oligocene - Rupelian | M-KS-Kąkolówka I |
| GIUS 10–3796/O/73 | 37x12 | Curved | Oligocene - Rupelian | M-KS-Kąkolówka I |
| GIUS 10–3796/O/74 | 30x29 | Irregular | Oligocene - Rupelian | M-KS-Kąkolówka I |
| GIUS 10–3796/O/75 | 50x32 | Irregular | Oligocene - Rupelian | M-KS-Kąkolówka I |
| GIUS 10–3796/O/76 | 37x15 | S-shaped | Oligocene - Rupelian | M-KS-Kąkolówka I |
| GIUS 10–3796/O/77 | 25x3 | Curved, Fig. 2g | Oligocene - Rupelian | M-KS-Kąkolówka I |
| GIUS 10–3796/O/78 | 25x4 | S-shaped | Oligocene - Rupelian | M-KS-Kąkolówka I |
| GIUS 10–3796/O/79 | 28x13 | Irregular | Oligocene - Rupelian | M-KS-Kąkolówka I |
| GIUS 10–3796/O/80 | 37x8 | Sinusoidal | Oligocene - Rupelian | M-KS-Kąkolówka I |
| GIUS 10–3796/O/81 | 28x20 | Irregular | Oligocene - Rupelian | M-KS-Kąkolówka I |
| GIUS 10–3796/O/82 | 24x9 | S-shaped | Oligocene - Rupelian | M-KS-Kąkolówka I |
| GIUS 10–3796/O/83 | 17x5 | Sinusoidal | Oligocene - Rupelian | M-KS-Kąkolówka I |
| GIUS 10–3796/O/84 | 24x6 | Sinusoidal | Oligocene - Rupelian | M-KS-Kąkolówka I |
| GIUS 10–3796/O/85 | 29x11 | Elongated | Oligocene - Rupelian | M-KS-Kąkolówka I |
| GIUS 10–3796/O/86 | 35x8 | Sinusoidal | Oligocene - Rupelian | M-KS-Kąkolówka I |
| GIUS 10–3796/O/87 | 44x10 | Irregular | Oligocene - Rupelian | M-KS-Kąkolówka I |
| GIUS 10–3796/O/88 | 41x15 | Curved | Oligocene - Rupelian | M-KS-Kąkolówka I |
| GIUS 10–3796/O/89 | 19x4 | Sinusoidal | Oligocene - Rupelian | M-KS-Kąkolówka I |
| GIUS 10–3796/O/90 | 25x6 | S-shaped | Oligocene - Rupelian | M-KS-Kąkolówka I |
| GIUS 10–3796/O/91 | 38x17 | Sinusoidal | Oligocene - Rupelian | M-KS-Kąkolówka I |
| GIUS 10–3796/O/92 | 34x12 | Curved | Oligocene - Rupelian | M-KS-Kąkolówka I |
| GIUS 10–3796/O/93 | 17x5 | S-shaped | Oligocene - Rupelian | M-KS-Kąkolówka I |
| GIUS 10–3796/O/94 | 32x16 | Irregular | Oligocene - Rupelian | M-KS-Kąkolówka I |
| GIUS 10–3796/O/95 | 37x5 | Curved | Oligocene - Rupelian | M-KS-Kąkolówka I |
| GIUS 10–3796/O/96 | 21x11 | Irregular | Oligocene - Rupelian | M-KS-Kąkolówka I |
| GIUS 10–3796/O/97 | 23x9 | Elongated | Oligocene - Rupelian | M-KS-Kąkolówka I |
| GIUS 10–3796/O/98 | 13x8 | Irregular, Fig. 2o | Oligocene - Rupelian | M-KS-Kąkolówka I |
| GIUS 10–3796/O/99 | 28x8 | Curved | Oligocene - Rupelian | M-KS-Kąkolówka I |
| GIUS 10–3796/O/100 | 30x19 | Irregular | Oligocene - Rupelian | M-KS-Kąkolówka I |
| GIUS 10–3796/O/101 | 34x10 | S-shaped | Oligocene - Rupelian | M-KS-Kąkolówka I |
| GIUS 10–3796/O/102 | 36x8 | Sinusoidal | Oligocene - Rupelian | M-KS-Kąkolówka I |
| GIUS 10–3796/O/103 | 40x13 | Curved | Oligocene - Rupelian | M-KS-Kąkolówka I |
| GIUS 10–3796/O/104 | 15x15 | Oval | Oligocene - Rupelian | M-KS-Kąkolówka I |
| GIUS 10–3796/O/105 | 17x9 | Sinusoidal | Oligocene - Rupelian | M-KS-Kąkolówka I |
| GIUS 10–3796/O/106 | 19x6 | Elongated | Oligocene - Rupelian | M-KS-Kąkolówka I |
| GIUS 10–3796/O/107 | 20x4 | S-shaped, Fig. 2p | Oligocene - Rupelian | M-KS-Kąkolówka I |
| GIUS 10–3796/O/108 | 25x4 | Curved | Oligocene - Rupelian | M-KS-Kąkolówka I |
| GIUS 10–3796/O/109 | 30x14 | Irregular | Oligocene - Rupelian | M-KS-Kąkolówka I |
| GIUS 10–3796/O/110 | 34x12 | Elongated | Oligocene - Rupelian | M-KS-Kąkolówka I |
| GIUS 10–3796/O/111 | 16x3 | S-shaped, Fig. 2r | Oligocene - Rupelian | M-KS-Kąkolówka I |
| GIUS 10–3796/O/112 | 18x7 | S-shaped | Oligocene - Rupelian | M-KS-Kąkolówka I |
| GIUS 10–3796/O/113 | 15x4 | Sinusoidal | Oligocene - Rupelian | M-KS-Kąkolówka I |
| GIUS 10–3796/O/114 | 37x10 | S-shaped | Oligocene - Rupelian | M-KS-Kąkolówka I |
| GIUS 10–3796/O/115 | 20x16 | Oval | Oligocene - Rupelian | M-KS-Kąkolówka I |
| GIUS 10–3796/O/116 | 32x9 | Sinusoidal | Oligocene - Rupelian | M-KS-Kąkolówka I |
| GIUS 10–3796/O/117 | 17x11 | Irregular | Oligocene - Rupelian | M-KS-Kąkolówka I |
| GIUS 10–3796/O/118 | 33x7 | Elongated | Oligocene - Rupelian | M-KS-Kąkolówka I |
| GIUS 10–3796/O/119 | 29x8 | Sinusoidal | Oligocene - Rupelian | M-KS-Kąkolówka I |
| GIUS 10–3796/O/120 | 22x6 | Curved | Oligocene - Rupelian | M-KS-Kąkolówka I |
| GIUS 10–3796/O/121 | 41x11 | Sinusoidal | Oligocene - Rupelian | M-KS-Kąkolówka I |
| GIUS 10–3796/O/122 | 31x13 | Elongated | Oligocene - Rupelian | M-KS-Kąkolówka I |
| GIUS 10–3796/O/123 | 12x10 | Oval | Oligocene - Rupelian | M-KS-Kąkolówka I |
| GIUS 10–3796/O/124 | 25x5 | Sinusoidal | Oligocene - Rupelian | M-KS-Kąkolówka I |
| GIUS 10–3796/O/125 | 27x4 | Sinusoidal | Oligocene - Rupelian | M-KS-Kąkolówka I |
| GIUS 10–3796/O/126 | 38x9 | S-shaped | Oligocene - Rupelian | M-KS-Kąkolówka I |
| GIUS 10–3796/O/127 | 35x7 | Sinusoidal | Oligocene - Rupelian | M-KS-Kąkolówka I |
| GIUS 10–3796/O/128 | 27x25 | Oval | Oligocene - Rupelian | M-KS-Kąkolówka I |
| GIUS 10–3796/O/129 | 35x10 | Sinusoidal | Oligocene - Rupelian | M-KS-Kąkolówka I |
| GIUS 10–3796/O/130 | 39x8 | Elongated | Oligocene - Rupelian | M-KS-Kąkolówka I |
| GIUS 10–3796/O/131 | 37x11 | Curved | Oligocene - Rupelian | M-KS-Kąkolówka I |
| GIUS 10–3796/O/132 | 34x6 | S-shaped | Oligocene - Rupelian | M-KS-Kąkolówka I |
| GIUS 10–3796/O/133 | 28x7 | Curved | Oligocene - Rupelian | M-KS-Kąkolówka I |
| GIUS 10–3796/O/134 | 30x11 | S-shaped | Oligocene - Rupelian | M-KS-Kąkolówka I |
| GIUS 10–3796/O/135 | 10x4 | S-shaped, Fig.2s | Oligocene - Rupelian | M-KS-Kąkolówka I |
| GIUS 10–3796/O/136 | 17x7 | Sinusoidal | Oligocene - Rupelian | M-KS-Kąkolówka I |
| GIUS 10–3796/O/137 | 20x6 | Elongated | Oligocene - Rupelian | M-KS-Kąkolówka I |
| GIUS 10–3796/O/138 | 22x13 | Irregular | Oligocene - Rupelian | M-KS-Kąkolówka I |
| GIUS 10–3796/O/139 | 40x25 | Oval, Fig. 2u | Oligocene - Rupelian | M-KS-Kąkolówka I |
| GIUS 10–3796/O/140 | 40x13 | S-shaped | Oligocene - Rupelian | M-KS-Kąkolówka I |
| GIUS 10–3796/O/141 | 37x20 | Irregular | Oligocene - Rupelian | M-KS-Kąkolówka I |
| GIUS 10–3796/O/142 | 31x15 | Irregular | Oligocene - Rupelian | M-KS-Kąkolówka I |
| GIUS 10–3796/O/143 | 33x13 | Curved | Oligocene - Rupelian | M-KS-Kąkolówka I |
| GIUS 10–3796/O/144 | 37x16 | Irregular, Fig. 3c | Oligocene - Rupelian | M-KS-Kąkolówka I |
| GIUS 10–3796/O/145 | 43x13 | S-shaped | Oligocene - Rupelian | M-KS-Kąkolówka I |
| GIUS 10–3796/O/146 | 40x10 | Sinusoidal | Oligocene - Rupelian | M-KS-Kąkolówka I |
| GIUS 10–3796/O/147 | 38x9 | Curved | Oligocene - Rupelian | M-KS-Kąkolówka I |
| GIUS 10–3796/O/148 | 23x10 | Elongated | Oligocene - Rupelian | M-KS-Kąkolówka I |
| GIUS 10–3796/O/149 | 29x6 | Elongated | Oligocene - Rupelian | M-KS-Kąkolówka I |
| GIUS 10–3796/O/150 | 17x14 | Oval | Oligocene - Rupelian | M-KS-Kąkolówka I |
| GIUS 10–3796/O/151 | 14x5 | Curved | Oligocene - Rupelian | M-KS-Kąkolówka II |
| GIUS 10–3796/O/152 | 30x8 | S-shaped | Oligocene - Rupelian | M-KS-Kąkolówka II |
| GIUS 10–3796/O/153 | 32x5 | S-shaped | Oligocene - Rupelian | M-KS-Kąkolówka II |
| GIUS 10–3796/O/154 | 26x2 | Sinusoidal, Fig. 2d | Oligocene - Rupelian | M-KS-Kąkolówka II |
| GIUS 10–3796/O/155 | 35x8 | Elongated | Oligocene - Rupelian | M-KS-Kąkolówka II |
| GIUS 10–3796/O/156 | 22x5 | Sinusoidal | Oligocene - Rupelian | M-KS-Kąkolówka II |
| GIUS 10–3796/O/157 | 32x7 | S-shaped | Oligocene - Rupelian | M-KS-Kąkolówka II |
| GIUS 10–3796/O/158 | 14x3 | Sinusoidal | Oligocene - Rupelian | M-KS-Kąkolówka II |
| GIUS 10–3796/O/159 | 8x6 | Oval | Oligocene - Rupelian | M-KS-Kąkolówka II |
| GIUS 10–3796/O/160 | 38x6 | Elongated | Oligocene - Rupelian | M-KS-Kąkolówka II |
| GIUS 10–3796/O/161 | 18x3 | Sinusoidal | Oligocene - Rupelian | M-KS-Kąkolówka II |
| GIUS 10–3796/O/162 | 15x8 | S-shaped | Oligocene - Rupelian | M-KS-Kąkolówka II |
| GIUS 10–3796/O/163 | 27x10 | Sinusoidal | Oligocene - Rupelian | M-KS-Kąkolówka II |
| GIUS 10–3796/O/164 | 38x21 | Irregular | Oligocene - Rupelian | M-KS-Kąkolówka II |
| GIUS 10–3796/O/165 | 24x9 | Sinusoidal | Oligocene - Rupelian | M-KS-Kąkolówka II |
| GIUS 10–3796/O/166 | 17x8 | Sinusoidal | Oligocene - Rupelian | M-KS-Kąkolówka II |
| GIUS 10–3796/O/167 | 33x6 | Elongated | Oligocene - Rupelian | M-KS-Kąkolówka II |
| GIUS 10–3796/O/168 | 39x8 | Curved | Oligocene - Rupelian | M-KS-Kąkolówka II |
| GIUS 10–3796/O/169 | 22x11 | Irregular | Oligocene - Rupelian | M-KS-Kąkolówka II |
| GIUS 10–3796/O/170 | 41x10 | Curved | Oligocene - Rupelian | M-KS-Kąkolówka II |
| GIUS 10–3796/O/171 | 35x13 | Elongated | Oligocene - Rupelian | M-KS-Kąkolówka II |
| GIUS 10–3796/O/172 | 12x4 | Sinusoidal | Oligocene - Rupelian | M-KS-Kąkolówka II |
| GIUS 10–3796/O/173 | 25x24 | Oval | Oligocene - Rupelian | M-KS-Kąkolówka II |
| GIUS 10–3796/O/174 | 37x17 | Elongated | Oligocene - Rupelian | M-KS-Kąkolówka II |
| GIUS 10–3796/O/175 | 15x12 | Sinusoidal | Oligocene - Rupelian | M-KS-Kąkolówka II |
| GIUS 10–3796/O/176 | 19x4 | Curved | Oligocene - Rupelian | M-KS-Kąkolówka II |
| GIUS 10–3796/O/177 | 19x6 | S-shaped | Oligocene - Rupelian | M-KS-Kąkolówka II |
| GIUS 10–3796/O/178 | 24x4 | Elongated | Oligocene - Rupelian | M-KS-Kąkolówka II |
| GIUS 10–3796/O/179 | 28x7 | Curved | Oligocene - Rupelian | M-KS-KąkolówkaII |
| GIUS 10–3796/O/180 | 30x8 | S-shaped | Oligocene - Rupelian | M-KS-Kąkolówka II |
| GIUS 10–3796/O/181 | 15x2 | Sinusoidal, Fig. 2e | Oligocene - Rupelian | M-KS-Kąkolówka II |
| GIUS 10–3796/O/182 | 33x11 | Curved | Oligocene - Rupelian | M-KS-Kąkolówka II |
| GIUS 10–3796/O/183 | 19x5 | Curved | Oligocene - Rupelian | M-KS-Kąkolówka II |
| GIUS 10–3796/O/184 | 20x7 | Sinusoidal | Oligocene - Rupelian | M-KS-Kąkolówka II |
| GIUS 10–3796/O/185 | 15x15 | Oval | Oligocene - Rupelian | M-KS-Kąkolówka II |
| GIUS 10–3796/O/186 | 37x8 | Sinusoidal | Oligocene - Rupelian | M-KS-Kąkolówka II |
| GIUS 10–3796/O/187 | 46x15 | Elongated | Oligocene - Rupelian | M-KS-Kąkolówka II |
| GIUS 10–3796/O/188 | 34x9 | Sinusoidal | Oligocene - Rupelian | M-KS-Kąkolówka II |
| GIUS 10–3796/O/189 | 18x11 | Elongated | Oligocene - Rupelian | M-KS-Kąkolówka II |
| GIUS 10–3796/O/190 | 33x6 | Curved | Oligocene - Rupelian | M-KS-Kąkolówka II |
| GIUS 10–3796/O/191 | 29x6 | Sinusoidal | Oligocene - Rupelian | M-KS-Kąkolówka II |
| GIUS 10–3796/O/192 | 23x7 | S-shaped | Oligocene - Rupelian | M-KS-Kąkolówka II |
| GIUS 10–3796/O/193 | 42x10 | Sinusoidal | Oligocene - Rupelian | M-KS-Kąkolówka II |
| GIUS 10–3796/O/194 | 37x13 | Sinusoidal | Oligocene - Rupelian | M-KS-Kąkolówka II |
| GIUS 10–3796/O/195 | 17x4 | Irregular | Oligocene - Rupelian | M-KS-Kąkolówka II |
| GIUS 10–3796/O/196 | 28x8 | Elongated | Oligocene - Rupelian | M-KS-Kąkolówka II |
| GIUS 10–3796/O/197 | 23x4 | Elongated | Oligocene - Rupelian | M-KS-Kąkolówka II |
| GIUS 10–3796/O/198 | 35x14 | Curved | Oligocene - Rupelian | M-KS-Kąkolówka II |
| GIUS 10–3796/O/199 | 38x13 | Curved | Oligocene - Rupelian | M-KS-Kąkolówka II |
| GIUS 10–3796/O/200 | 40x17 | S-shaped | Oligocene - Rupelian | M-KS-Kąkolówka II |
| GIUS 10–3796/O/201 | 44x10 | Elongated | Oligocene - Rupelian | M-KS-Kąkolówka II |
| GIUS 10–3796/O/202 | 17x14 | Oval | Oligocene - Rupelian | M-KS-Kąkolówka II |
| GIUS 10–3796/O/203 | 35x12 | S-shaped | Oligocene - Rupelian | M-KS-Kąkolówka II |
| GIUS 10–3796/O/204 | 17x6 | Sinusoidal | Oligocene - Rupelian | M-KS-Kąkolówka II |
| GIUS 10–3796/O/205 | 20x7 | S-shaped | Oligocene - Rupelian | M-KS-Kąkolówka II |
| GIUS 10–3796/O/206 | 18x9 | S-shaped | Oligocene - Rupelian | M-KS-Kąkolówka II |
| GIUS 10–3796/O/207 | 22x9 | Elongated | Oligocene - Rupelian | M-KS-Kąkolówka II |
| GIUS 10–3796/O/208 | 26x8 | Sinusoidal | Oligocene - Rupelian | M-KS-Kąkolówka II |
| GIUS 10–3796/O/209 | 38x9 | Curved | Oligocene - Rupelian | M-KS-Kąkolówka II |
| GIUS 10–3796/O/210 | 24x9 | Sinusoidal | Oligocene - Rupelian | M-KS-Kąkolówka II |
| GIUS 10–3796/O/211 | 17x15 | Oval | Oligocene - Rupelian | M-KS-Kąkolówka II |
| GIUS 10–3796/O/212 | 23x12 | Irregular | Oligocene - Rupelian | M-KS-Kąkolówka II |
| GIUS 10–3796/O/213 | 31x8 | Sinusoidal | Oligocene - Rupelian | M-KS-Kąkolówka II |
| GIUS 10–3796/O/214 | 35x11 | Irregular | Oligocene - Rupelian | M-KS-Kąkolówka II |
| GIUS 10–3796/O/215 | 39x13 | Sinusoidal | Oligocene - Rupelian | M-KS-Kąkolówka II |
| GIUS 10–3796/O/216 | 44x9 | Sinusoidal | Oligocene - Rupelian | M-KS-Kąkolówka II |
| GIUS 10–3796/O/217 | 12x4 | Elongated | Oligocene - Rupelian | M-KS-Kąkolówka II |
| GIUS 10–3796/O/218 | 25x7 | S-shaped | Oligocene - Rupelian | M-KS-Kąkolówka II |
| GIUS 10–3796/O/219 | 29x14 | Sinusoidal | Oligocene - Rupelian | M-KS-Kąkolówka II |
| GIUS 10–3796/O/220 | 37x13 | S-shaped | Oligocene - Rupelian | M-KS-Kąkolówka II |
| GIUS 10–3796/O/221 | 41x17 | Sinusoidal | Oligocene - Rupelian | M-KS-Kąkolówka II |
| GIUS 10–3796/O/222 | 47x11 | Sinusoidal | Oligocene - Rupelian | M-KS-Kąkolówka II |
| GIUS 10–3796/O/223 | 52x20 | Sinusoidal | Oligocene - Rupelian | M-KS-Kąkolówka II |
| GIUS 10–3796/O/224 | 31x14 | Elongated | Oligocene - Rupelian | M-KS-Kąkolówka II |
| GIUS 10–3796/O/225 | 27x26 | Oval | Oligocene - Rupelian | M-KS-Kąkolówka II |
| GIUS 10–3796/O/226 | 36x13 | Elongated | Oligocene - Rupelian | M-KS-Kąkolówka II |
| GIUS 10–3796/O/227 | 38x15 | S-shaped | Oligocene - Rupelian | M-KS-Kąkolówka II |
| GIUS 10–3796/O/228 | 16x5 | S-shaped | Oligocene - Rupelian | M-KS-Kąkolówka II |
| GIUS 10–3796/O/229 | 25x8 | Sinusoidal | Oligocene - Rupelian | M-KS-Kąkolówka II |
| GIUS 10–3796/O/230 | 20x4 | Elongated | Oligocene - Rupelian | M-KS-Kąkolówka II |
| GIUS 10–3796/O/231 | 27x10 | S-shaped | Oligocene - Rupelian | M-KS-Kąkolówka II |
| GIUS 10–3796/O/232 | 26x25 | Oval | Oligocene - Rupelian | M-KS-Kąkolówka II |
| GIUS 10–3796/O/233 | 38x17 | Sinusoidal | Oligocene - Rupelian | M-KS-Kąkolówka II |
| GIUS 10–3796/O/234 | 23x11 | Curved | Oligocene - Rupelian | M-KS-Kąkolówka II |
| GIUS 10–3796/O/235 | 17x5 | Elongated | Oligocene - Rupelian | M-KS-Kąkolówka II |
| GIUS 10–3796/O/236 | 23x12 | Irregular | Oligocene - Rupelian | M-KS-Kąkolówka II |
| GIUS 10–3796/O/237 | 32x9 | Sinusoidal | Oligocene - Rupelian | M-KS-Kąkolówka II |
| GIUS 10–3796/O/238 | 35x11 | Curved | Oligocene - Rupelian | M-KS-Kąkolówka II |
| GIUS 10–3796/O/239 | 39x12 | Elongated | Oligocene - Rupelian | M-KS-Kąkolówka II |
| GIUS 10–3796/O/240 | 54x10 | S-shaped | Oligocene - Rupelian | M-KS-Kąkolówka II |
| GIUS 10–3796/O/241 | 13x4 | Sinusoidal | Oligocene - Rupelian | M-KS-Kąkolówka II |
| GIUS 10–3796/O/242 | 21x7 | Curved | Oligocene - Rupelian | M-KS-Kąkolówka II |
| GIUS 10–3796/O/243 | 29x14 | Elongated | Oligocene - Rupelian | M-KS-Kąkolówka II |
| GIUS 10–3796/O/244 | 12x3 | Elongated | Oligocene - Rupelian | M-KS-Kąkolówka II |
| GIUS 10–3796/O/245 | 36x13 | Elongated | Oligocene - Rupelian | M-KS-Kąkolówka II |
| GIUS 10–3796/O/246 | 28x11 | Irregular | Oligocene - Rupelian | M-KS-Kąkolówka II |
| GIUS 10–3796/O/247 | 56x14 | Sinusoidal | Oligocene - Rupelian | M-KS-Kąkolówka II |
| GIUS 10–3796/O/248 | 13x11 | Oval | Oligocene - Rupelian | M-KS-Kąkolówka II |
| GIUS 10–3796/O/249 | 22x7 | Curved | Oligocene - Rupelian | M-KS-Kąkolówka II |
| GIUS 10–3796/O/250 | 29x16 | Curved | Oligocene - Rupelian | M-KS- Kąkolówka II |
| GIUS 10–3796/O/251 | 18x2 | Elongated, Fig. 2h | Oligocene - Rupelian | M-KS-Wola Czudecka |
| GIUS 10–3796/O/252 | 39x15 | elongated | Oligocene - Rupelian | M-KS-Wola Czudecka |
| GIUS 10–3796/O/253 | 12x2 | Elongated, Fig. 2i | Oligocene - Rupelian | M-KS-Wola Czudecka |
| GIUS 10–3796/O/254 | 19x4 | S-shaped | Oligocene - Rupelian | M-KS-Wola Czudecka |
| GIUS 10–3796/O/255 | 28x10 | Sinusoidal | Oligocene - Rupelian | M-KS-Wola Czudecka |
| GIUS 10–3796/O/256 | 12x3 | Irregular; Fig. 2n | Oligocene - Rupelian | M-KS-Wola Czudecka |
| GIUS 10–3796/O/257 | 37x8 | S-shaped | Oligocene - Rupelian | M-KS-Wola Czudecka |
| GIUS 10–3796/O/258 | 39x11 | Curved | Oligocene - Rupelian | M-KS-Wola Czudecka |
| GIUS 10–3796/O/259 | 11x2 | Elongated, Fig. 2j | Oligocene - Rupelian | M-KS-Wola Czudecka |
| GIUS 10–3796/O/260 | 39x9 | Elongated | Oligocene - Rupelian | M-KS-Wola Czudecka |
| GIUS 10–3796/O/261 | 36x13 | Sinusoidal | Oligocene - Rupelian | M-KS-Wola Czudecka |
| GIUS 10–3796/O/262 | 24x24 | Oval | Oligocene - Rupelian | M-KS-Wola Czudecka |
| GIUS 10–3796/O/263 | 15x7 | Curved | Oligocene - Rupelian | M-KS-Wola Czudecka |
| GIUS 10–3796/O/264 | 26x5 | Sinusoidal | Oligocene - Rupelian | M-KS-Wola Czudecka |
| GIUS 10–3796/O/265 | 33x14 | Irregular | Oligocene - Rupelian | M-KS-Wola Czudecka |
| GIUS 10–3796/O/266 | 14x4 | Curved | Oligocene - Rupelian | M-KS-Wola Czudecka |
| GIUS 10–3796/O/267 | 36x13 | Sinusoidal | Oligocene - Rupelian | M-KS-Wola Czudecka |
| GIUS 10–3796/O/268 | 38x11 | Elongated | Oligocene - Rupelian | M-KS-Wola Czudecka |
| GIUS 10–3796/O/269 | 50x22 | S-shaped | Oligocene - Rupelian | M-KS-Wola Czudecka |
| GIUS 10–3796/O/270 | 12x13 | Sinusoidal | Oligocene - Rupelian | M-KS-Wola Czudecka |
| GIUS 10–3796/O/271 | 28x7 | Elongated | Oligocene - Rupelian | M-KS-Wola Czudecka |
| GIUS 10–3796/O/272 | 35x14 | Curved | Oligocene - Rupelian | M-KS-Wola Czudecka |
| GIUS 10–3796/O/273 | 21x18 | Oval | Oligocene - Rupelian | M-KS-Wola Czudecka |
| GIUS 10–3796/O/274 | 20x3 | Elongated, Fig. 2k | Oligocene - Rupelian | M-KS-Wola Czudecka |
| GIUS 10–3796/O/275 | 42x16 | Elongated | Oligocene - Rupelian | M-KS-Wola Czudecka |
| GIUS 10–3796/O/276 | 38x5 | Sinusoidal | Oligocene - Rupelian | M-KS-Wola Czudecka |
| GIUS 10–3796/O/277 | 39x9 | S-shaped | Oligocene - Rupelian | M-KS-Wola Czudecka |
| GIUS 10–3796/O/278 | 41x13 | Sinusoidal | Oligocene - Rupelian | M-KS-Futoma |
| GIUS 10–3796/O/279 | 11x7 | Oval, Fig. 2l | Oligocene - Rupelian | M-KS-Futoma |
| GIUS 10–3796/O/280 | 11x4 | Elongated | Oligocene - Rupelian | M-KS-Futoma |
| GIUS 10–3796/O/281 | 19x6 | S-shaped | Oligocene - Rupelian | M-KS-Futoma |
| GIUS 10–3796/O/282 | 12x4 | Irregular, Fig. 2m | Oligocene - Rupelian | M-KS-Futoma |
| GIUS 10–3796/O/283 | 33x9 | Sinusoidal | Oligocene - Rupelian | M-KS-Futoma |
| GIUS 10–3796/O/284 | 35x11 | Curved | Oligocene - Rupelian | M-KS-Futoma |
| GIUS 10–3796/O/285 | 32x13 | Elongated | Oligocene - Rupelian | M-KS-Wujskie |
| GIUS 10–3796/O/286 | 20x7 | Sinusoidal | Oligocene - Rupelian | M-KS-Wujskie |
| GIUS 10–3796/O/287 | 26x5 | Sinusoidal | Oligocene - Rupelian | M-KS-Wujskie |
| GIUS 10–3796/O/288 | 11x3 | Elongated | Oligocene - Rupelian | M-KS-Wujskie |
| GIUS 10–3796/O/289 | 19x4 | Elongated | Oligocene - Rupelian | M-KS-Rudawka Rymanowska |
| GIUS 10–3796/O/290 | 45x15 | Sinusoidal | Oligocene - Rupelian | M-KS-Rudawska Rymanowska |
| GIUS 10–3796/O/291 | 28x15 | Sinusoidal | Oligocene - Rupelian | M-KS-Rudawka Rymanowska |
| GIUS 10–3796/O/292 | 14x11 | Oval | Oligocene - Rupelian | M-KS-Jamna Dolna |
| GIUS 10–3796/O/293 | 57x10 | Curved | Oligocene - Rupelian | M-KS-Jamna Dolna |
| GIUS 10–3796/O/294 | 9x4 | Elongated, Fig. 2t | Oligocene - Rupelian | M-KS-Jamna Dolna |
| GIUS 10–3796/O/295 | 19x13 | Irregular | Oligocene - Rupelian | M-KS-Jamna Dolna |
| GIUS 10–3796/O/296 | 22x7 | Elongated | Oligocene - Rupelian | M-KS-Jamna Dolna |
| GIUS 10–3796/O/297 | 9x7 | Oval, Fig. 3a | Oligocene - Rupelian | M-KS-Równe |
| GIUS 10–3796/O/298 | 13x13 | Oval | Oligocene - Rupelian | M-KS-Równe |
| GIUS 10–3796/O/299 | 25x6 | Sinusoidal, Fig.3b | Oligocene - Rupelian | M-KS-Jasienica Rosielna |
| GIUS 10–3796/O/300 | 20x4 | Curved | Oligocene - Rupelian | M-KS-Jasienica Rosielna |
