# Supplementary material for: From trace to trace maker: Oligocene–Miocene coprolites of southern Poland and their potential producers
Source: PeerJ. 2025 Nov 3;13:e20242. doi: 10.7717/peerj.20242 (PMC12591054; doi:10.7717/peerj.20242)
Supplement: Supplemental Information 9 [file peerj-13-20242-s009.docx]

**Table 2:**

**Miocene coprolite list.**

| **Specimen** | **Dimensions (mm)** | **Shape** | **Age** | **Site** |
| --- | --- | --- | --- | --- |
| GIUS 10–3796/M/1 | 36x28 | Oval | Miocene - Langhian | Kleszczów Graben area-Bełchatów |
| GIUS 10–3796/M/2 | 25x15 | Irregular, Fig. 3l | Miocene - Langhian | Kleszczów Graben area-Bełchatów |
| GIUS 10–3796/M/3 | 19x17 | Oval | Miocene - Langhian | Kleszczów Graben area-Bełchatów |
| GIUS 10–3796/M/4 | 21x17 | Oval | Miocene - Langhian | Kleszczów Graben area-Bełchatów |
| GIUS 10–3796/M/5 | 27x8 | Curved | Miocene - Langhian | Kleszczów Graben area-Bełchatów |
| GIUS 10–3796/M/6 | 10x5 | Elongated, Fig. 3m | Miocene - Langhian | Kleszczów Graben area-Bełchatów |
| GIUS 10–3796/M/6(1) | 18x8 | Elongated | Miocene - Langhian | Kleszczów Graben area-Bełchatów |
| GIUS 10–3796/M/6(2) | 16x10 | Elongated | Miocene - Langhian | Kleszczów Graben area-Bełchatów |
| GIUS 10–3796/M/6(3) | 19x11 | Elongated | Miocene - Langhian | Kleszczów Graben area-Bełchatów |
| GIUS 10–3796/M/6(4) | 15x8 | Elongated | Miocene - Langhian | Kleszczów Graben area-Bełchatów |
| GIUS 10–3796/M/6(5) | 19x12 | Elongated | Miocene - Langhian | Kleszczów Graben area-Bełchatów |
| GIUS 10–3796/M/7 | 27x25 | Oval | Miocene - Langhian | Kleszczów Graben area-Bełchatów |
| GIUS 10–3796/M/8 | 37x13 | Elongated | Miocene - Langhian | Kleszczów Graben area-Bełchatów |
| GIUS 10–3796/M/9 | 47x18 | Irregular | Miocene - Langhian | Kleszczów Graben area-Bełchatów |
| GIUS 10–3796/M/10 | 31x30 | Oval | Miocene - Langhian | Kleszczów Graben area-Bełchatów |
| GIUS 10–3796/M/11 | 20x17 | Oval, Fig. 3n | Miocene - Langhian | Kleszczów Graben area-Bełchatów |
| GIUS 10–3796/M/12 | 16x5 | Sinusoidal | Miocene - Langhian | Kleszczów Graben area-Bełchatów |
| GIUS 10–3796/M/13 | 20x8 | Elongated, Fig. 3e | Miocene - Langhian | Gołuchów quarry |
| GIUS 10–3796/M/14 | 40x14 | S-shaped | Miocene - Burdigalian | Turów area |
| GIUS 10–3796/M/15 | 61x24 | Curved | Miocene - Burdigalian | Turów area |
| GIUS 10–3796/M/16 | 65x28 | Elongated, Fig. 3g | Miocene - Burdigalian | Turów area |
| GIUS 10–3796/M/17 | 34x24 | Oval | Miocene - Burdigalian | Turów area |
| GIUS 10–3796/M/18 | 41x13 | S-shaped | Miocene - Burdigalian | Turów area |
| GIUS 10–3796/M/19 | 50x30 | Curved, Fig. 3h | Miocene - Burdigalian | Turów area |
| GIUS 10–3796/M/20 | 36x18 | Elongated | Miocene - Burdigalian | Turów area |
| GIUS 10–3796/M/21 | 40x14 | Sinusoidal | Miocene - Burdigalian | Turów area |
| GIUS 10–3796/M/22 | 31x10 | S-shaped | Miocene - Burdigalian | Turów area |
| GIUS 10–3796/M/23 | 42x28 | Irregular, Fig. 3i | Miocene - Burdigalian | Turów area |
| GIUS 10–3796/M/24 | 54x23 | Sinusoidal | Miocene - Burdigalian | Turów area |
| GIUS 10–3796/M/25 | 36x16 | Sinusoidal | Miocene - Burdigalian | Turów area |
| GIUS 10–3796/M/26 | 30x30 | Oval | Miocene - Burdigalian | Turów area |
| GIUS 10–3796/M/27 | 48x19 | Elongated | Miocene - Burdigalian | Turów area |
| GIUS 10–3796/M/28 | 58x27 | Curved, Fig.3j | Miocene - Burdigalian | Turów area |
| GIUS 10–3796/M/29 | 73x25 | Elongated | Miocene - Burdigalian | Turów area |
| GIUS 10–3796/M/20 | 48x20 | S-shaped, Fig. 3k | Miocene - Burdigalian | Turów area |
| GIUS 10–3796/M/31 | 41x13 | Curved | Miocene - Burdigalian | Turów area |
| GIUS 10–3796/M/32 | 21x10 | Elongated, Fig. 3f | Miocene - Serravalian | Roztocze area-Żelebsko |
| GIUS 10–3796/M/33 | 19x5 | Elongated, Fig. 3d | Miocene - Burdigalian | M-KS-Temeszów |
| GIUS 10–3796/M/34 | 30x10 | Elongated | Miocene - Burdigalian | M-KS-Brzuska |
