# Supplementary material for: From trace to trace maker: Oligocene–Miocene coprolites of southern Poland and their potential producers
Source: PeerJ. 2025 Nov 3;13:e20242. doi: 10.7717/peerj.20242 (PMC12591054; doi:10.7717/peerj.20242)
Supplement: Supplemental Information 10 [file peerj-13-20242-s010.docx]

**Table 3:**

**Oligocene localities with coprolites and their morphologies.**

|  | **SHAPE** | | | | | |  |
| --- | --- | --- | --- | --- | --- | --- | --- |
| **Locality** | **Sinusoidal** | **Elongated** | **Oval** | **More or less regular** | **S-shaped** | **Curved** | **Summary** |
| M-KS-Kąkolówka I | 40 | 23 | 18 | 17 | 22 | 30 | 150 |
| M-KS-Kąkolówka II | 34 | 21 | 8 | 7 | 17 | 13 | 100 |
| M-KS-Wola Czudecka | 6 | 9 | 2 | 1 | 4 | 5 | 27 |
| M-KS-Futoma | 2 | 1 | 1 | 1 | 1 | 1 | 7 |
| M-KS-Jamna Dolna | - | 2 | 1 | 1 | - | 1 | 5 |
| M-KS-Rudawka Rymanowska | 2 | 1 | - | - | - | - | 3 |
| M-KS-Równe | - | - | 2 | - | - | - | 2 |
| M-KS-Wujskie | 2 | 2 | - | - | - | - | 4 |
| M-KS-Jasienica Rosielna | 1 | - | - | - | - | 1 | 2 |
