# Supplementary material for: From trace to trace maker: Oligocene–Miocene coprolites of southern Poland and their potential producers
Source: PeerJ. 2025 Nov 3;13:e20242. doi: 10.7717/peerj.20242 (PMC12591054; doi:10.7717/peerj.20242)
Supplement: Supplemental Information 11 [file peerj-13-20242-s011.docx]

**Table 4:**

**Miocene localities with coprolites and their morphologies.**

|  | **SHAPE** | | | | | |  |
| --- | --- | --- | --- | --- | --- | --- | --- |
| **Locality** | **Sinusoidal** | **Elongated** | **Oval** | **More or less regular** | **S-shaped** | **Curved** | **Summary** |
| Kleszczów Graben area | 5 | 9 | 2 | 1 | - | 1 | 17 |
| Turów area | 3 | 4 | 2 | 1 | 4 | 4 | 18 |
| Gołuchów quarry | - | 1 | - | - | - | - | 1 |
| Roztocze area-Żelebsko |  |  |  |  |  |  | 1 |
| M-KS-Temeszów | - | 1 | - | - | - | - | 1 |
| M-KS-Brzuska | - | 1 | - | - | - | - | 1 |
